# Long-Term Hypoxia Negatively Influences Ca^2+^ Signaling in Basilar Arterial Myocytes of Fetal and Adult Sheep

**DOI:** 10.3389/fphys.2021.760176

**Published:** 2022-01-18

**Authors:** Casey Reid, Monica Romero, Stephanie B. Chang, Noah Osman, Jose L. Puglisi, Christopher G. Wilson, Arlin B. Blood, Lubo Zhang, Sean M. Wilson

**Affiliations:** ^1^Lawrence D. Longo, MD Center for Perinatal Biology, Loma Linda University School of Medicine, Loma Linda, CA, United States; ^2^Advanced Imaging and Microscopy Core, Loma Linda University School of Medicine, Loma Linda, CA, United States; ^3^Department of Biostatistics, School of Medicine, California Northstate University, Elk Grove, CA, United States

**Keywords:** arterial myocytes, calcium oscillations in living cells, calcium sparks, ontogeny, high altitude

## Abstract

Cerebral arterial vasoreactivity is vital to the regulation of cerebral blood flow. Depolarization of arterial myocytes elicits whole-cell Ca^2+^ oscillations as well as subcellular Ca^2+^ sparks due to activation of ryanodine receptors on the sarcoplasmic reticulum. Previous evidence illustrates that contraction of cerebral arteries from sheep and underlying Ca^2+^ signaling pathways are modified by age and that long-term hypoxia (LTH) causes aberrations in Ca^2+^ signaling pathways and downstream effectors impacting vasoregulation. We hypothesize that age and LTH affect the influence of membrane depolarization on whole-cell intracellular Ca^2+^ oscillations and sub-cellular Ca^2+^ spark activity in cerebral arteries. To test this hypothesis, we examined Ca^2+^ oscillatory and spark activities using confocal fluorescence imaging techniques of Fluo-4 loaded basilar arterial myocytes of low- and high-altitude term fetal (∼145 days of gestation) and adult sheep, where high-altitude pregnant and non-pregnant sheep were placed at 3,801 m for >100 days. Ca^2+^ oscillations and sparks were recorded using an *in situ* preparation evaluated in the absence or presence of 30 mM K^+^ (30K) to depolarize myocytes. Myocytes from adult animals tended to have a lower basal rate of whole-cell Ca^2+^ oscillatory activity and 30K increased the activity within cells. LTH decreased the ability of myocytes to respond to depolarization independent of age. These observations illustrate that both altitude and age play a role in affecting whole-cell and localized Ca^2+^ signaling, which are important to arterial vasoreactivity and cerebral blood flow.

## Introduction

In an age where cardiovascular health is so heavily scrutinized, we still struggle to reduce deaths from cardiovascular diseases. With the identification of modifiable risk factors such as smoking, diet, exercise, and obesity, we are now able to help prevent a significant portion of the morbidity and mortality associated with cardiovascular disease; however, environmental exposures that we have little or no control over are important to cardiovascular development and health (Yang et al., 2017). What is more, previous research illustrates that many cardiovascular diseases have origins in fetal development and that gestational stressors have complex effects and impacts on vascular health (Ducsay et al., 2018).

Gestational long-term hypoxia (LTH) from living at high altitude or placental insufficiency of various etiologies has a marked effect on fetal cerebral vascular development that can have repercussions throughout an individual’s lifetime (Ducsay et al., 2018). Decreasing oxygenation during the fetal period plays a significant role in vascular development, affecting vessel structure and function. Low oxygen exposures cause significant changes in the regulation of cellular phenotype, which influences vasoreactivity (Adeoye et al., 2015; Ducsay et al., 2018). Cerebral vascular dysfunction can result in an increased risk of cerebral edema, hemorrhage, and ischemic stroke. Furthermore, there are correlations between brain blood flow, developmental neuropathies, and impaired learning in children as well as psychological disorders such as clinical depression (Koenigs and Grafman, 2009) and dementia (Roher et al., 2012). These interrelationships illustrate the importance of considering the role of dysregulated vessel structure and function in neurological disorders.

Traveling to high altitude has significant effects on cerebrovascular blood flow (CBF). Cerebral arteries dilate in response to acute hypoxia, allowing for distribution of a greater portion of cardiac output to the brain in an attempt to maintain oxygen delivery despite reduced oxygen tensions (Hunter et al., 2003; Lee et al., 2009; Giussani, 2016). However, the reduced cerebral arterial contractility impairs the autoregulation of CBF (Tweed et al., 1983, 1986) and increases the risk of hemorrhage of the germinal matrix leading to varying brain disorders including epilepsy, cerebral palsy, and intellectual disability (Volpe, 1997; Nelson, 2003; Ferriero, 2004; Stoll et al., 2010). Vasodilation of cerebral vessels in response to acute hypoxia is partially regulated by an increase in large conductance potassium (BK_Ca_) channel activation, which is responsible for membrane hyperpolarization and consequent vasorelaxation (Gebremedhin et al., 1994; Brenner et al., 2000; Ledoux et al., 2006; Thorpe et al., 2017). Long-term hypoxia, in comparison to acute hypoxia, can lead to compensatory responses that reduce CBF toward control levels. Studies done on adult animals as well as humans show that a large component of this compensation is due to increased ventilation and hematocrit, both of which serve to maintain cerebral O_2_ delivery (Brugniaux et al., 2007). Prior to these adaptations and acclimatization, there is a correlation between hypoxia and increased basilar blood flow due to increases in arterial diameter (Jansen et al., 2002; Ainslie and Subudhi, 2014). Studies in fetal animals have shown that gestational LTH leads to a sustained increase in the proportion of cardiac output serving the brain (Ducsay et al., 2018). However, there is an impaired ability to increase cerebral blood flow in response to superimposed acute hypoxia (Pena et al., 2007), suggestive of altered cerebral vasoactivity. This is consistent with evidence that chronic hypoxia alters many of the pathways that regulate calcium signaling, and thus vascular tone, in fetal cerebral arteries (Pearce, 2006).

The current series of studies were designed to interrogate the effects of animal age and long-term hypoxia on calcium signals in basilar arterial myocytes under resting conditions and in response to membrane depolarization. We hypothesized that long-term hypoxia due to high altitude exposure would alter calcium signaling in ways that may restrict BK_Ca_ channel activation. We theorized that in arteries from low altitude fetuses, myocyte depolarization would increase whole-cell Ca^2+^ oscillations and localized rapid calcium signals (Ca^2+^ sparks) but that LTH would blunt these responses. We further hypothesized that post-natal development would magnify the increases in whole-cell and localized Ca^2+^ responses to membrane depolarization. This was examined by interrogating Ca^2+^ signals in basilar cerebral arterial myocytes of fetal and adult sheep housed at low- or high-altitude.

## Materials and Methods

### Experimental Animals

Surgical and experimental procedures were performed in accordance with the regulations of the Animal Welfare Act, the National Institutes of Health’s Guide for the Care and Use of Laboratory Animals, and “The Guiding Principles in the Care and Use of Animals” approved by the Council of the American Physiological Society and by the Institutional Animal Care and Use Committee of Loma Linda University. Pregnant (*n* = 7) and non-pregnant ewes (*n* = 7) of a mixed Western breed were divided into low altitude (normoxic) and high-altitude long-term hypoxic (LTH) groups. All ewes were obtained from Nebeker Ranch in Lancaster, CA, United States at an elevation near sea level, 720 m. Normoxic control ewes were maintained near sea level at 720 m for the duration of their gestation. Animals for the LTH groups were held at Nebeker Ranch under normoxic conditions until 30 days gestation at which time the pregnant and non-pregnant ewes were transported to the Barcroft Laboratory, White Mountain Research Station in Bishop, CA, United States at an elevation of 3,801 m. Previous work shows that residing at the Barcroft laboratory results in a maternal arterial PO_2_ of 60 ± 3 Torr and a fetal arterial PO_2_ of 19 ± 2 Torr (Kamitomo et al., 1993). Animals were held at elevation for the remaining ∼110 days of gestation for pregnant ewes and acclimatization for the non-pregnant ewes. Following this acclimatization period, ewes were transported (∼6 h drive) to Loma Linda University (LLU) for study at an elevation of 346 m. Once at LLU, LTH ewes were surgically instrumented with arterial and tracheal catheters. Based on frequent arterial blood gas sampling and adjustment of the rate of N_2_ flow through the tracheal catheter, arterial PO_2_ level in the adult sheep was maintained at ∼60 Torr for 2–4 days, mimicking the conditions of the effects of high altitude until the day of study (Kamitomo et al., 1993). After induction with thiopental sodium (10 mg/kg iv), the ewes were intubated and anesthesia was maintained via inhalation of 2–3% isoflurane in O_2_ for the duration of the surgery. The fetuses were delivered via hysterectomy at a male to female ratio of ∼1:1. Following delivery, fetal sheep were euthanized with an overdose of Euthasol (pentobarbital sodium, 100 mg/kg) and phenytoin sodium (10 mg/kg).

### Artery Isolation

Non-pregnant females and mixed sex, near term fetal brains were removed and placed in iced Balanced Salt Solution (BSS) of the following composition (mM): 126 NaCl; 5 KCl; 10 HEPES; 1 MgCl_2_; 2 CaCl_2_; 10 glucose; pH 7.4 (adjusted with NaOH). Basilar arteries then were quickly dissected under normoxic conditions in BSS. Basilar arteries were selected from the same anatomical locations in both fetal and adult sheep to maintain segments of similar function and embryological origin. Because of this, there was a significant difference in diameter between fetal and adult arteries (∼200 μm vs. 300 μm, respectively) as described previously (Lin et al., 2003; Tao et al., 2015). All experiments were performed under normoxic conditions at room temperature (∼22–24°C).

### Cytosolic Ca^2+^ Imaging

Intracellular Ca^2+^ of basilar arterial myocytes was measured *in situ* with a Ca^2+^ sensitive fluorescent dye (Fluo-4 AM, Cat No F14201, Invitrogen, Carlsbad, CA, United States) using a Zeiss 710 NLO laser scanning confocal imaging work station (Thornwood, NY, United States) with an inverted microscope (ZEISS Axio Observer), using procedures based on previous studies (Hadley et al., 2012; Harraz et al., 2014; Shen et al., 2018). Fluo-4 AM was dissolved in DMSO creating a 1 mM stock solution. Arteries were placed in BSS and exposed to a Fluo-4 concentration of 10 μM with 0.1% Pluronic F-127 Cat No P6867 (Invitrogen) from a 20% w/v stock solution in DMSO for 1 h in the dark at room temperature. These arterial segments were subsequently washed with BSS for 30 min to facilitate dye esterification and were then cut into linear strips for testing. These strips were pinned to Sylgard *en face* (Ellsworth Adhesives, Germantown, WI, United States) with fine insect dissecting pins and placed into an open bath imaging chamber (Warner Instruments, Hamden, CT, United States). Myocytes were illuminated at 488 nm via a Krypton-argon laser. Emitted light was captured with a photomultiplier tube with a band limited spectral grating of range 493–622 nm in both full frame and line scan imaging studies.

### Whole-Cell Ca^2+^ Recordings

A time series of 300 full frame Fluo-4 fluorescence images of 512 × 512 pixels were made over 234 s (roughly 780 ms/frame). In order to make sure that the smooth muscle intracellular Ca^2+^ was recorded, the pinhole was set at an imaging depth of 5.4 μm, which is roughly the depth of an individual smooth muscle cell based on examination of both fixed and live preparations (Hadley et al., 2012; Shen et al., 2018). This thicker optical sectioning of samples was performed to mitigate the effects of sample ruffling, allowing for visualization of more myocytes than would otherwise be possible at other depths. The sample was focused just below the internal elastic lamina to center on the myocytes and avoid the significant autofluorescence of this layer when excited at 488 nm. Images were taken using 12-bit sampling and a water immersion 63X Plan Apochromat, 1.4 NA objective. Under basal conditions a time series was made to assess the Ca^2+^ oscillatory behavior of individual myocytes. After the video recording 30–50 line scans of 18.9 s were collected as detailed below, which were used to measure Ca^2+^ spark activity. Careful effort was made to not duplicate imaging regions for the line scans, which ensured proper sampling of individual cells in the arterial wall and reduced the potential for photobleaching and laser induced toxicity. Following basal recordings, BSS with 30 mM K (30K) through equimolar replacement of potassium for sodium was added to the tissues to depolarize the plasma membrane of the myocytes (Hadley et al., 2012; Harraz et al., 2014; Shen et al., 2018). 5 min later we performed another time series recording, followed by series of line scan recordings.

### Whole-Cell Ca^2+^ Analysis

Regions of interest for full frame fluorescent imaging were automatically detected using the LCPro plug in for Fiji (ImageJ) (Francis et al., 2012; Schindelin et al., 2012), following 8 bit image conversion, image registration with StackReg using rigid body settings and cropping the images to 490 × 490 pixels to remove any erroneous pixels due to image registration (Thévenaz et al., 1998; Francis et al., 2012; Shen et al., 2018). LCPro was used to calculate fractional fluorescence intensity for automatically detected regions of interest, based on user defined region sizes, which were circles of 1.56 μm (six pixels). This region size was chosen as it provides analysis within individual cells as opposed to larger regions, which often overlap with adjacent cells.

The program performs analysis of the fluorescent intensity increases for each of the detected regions of interest above statistical noise, which was set to a threshold of *P* < 0.05. The program then analyzes the signal in the time and space domains for several parameters. In the current study, examinations were made of the following parameters: Amplitude (F/F_0_), which is the maximum amplitude of the signal transient as defined by the global maximum of the F/F_0_ curve within the time-period of the signal. Duration (s), which is the time interval defined by the period from one-half of the maximal amplitude values before and after the signal peak. Rise Time (s), which is written as “attack” in the program output and is the time interval defined by the period from one-half of the maximal signal amplitude value preceding the signal peak to the signal peak. Decay (s), which is the time interval defined by the time at the maximum amplitude to the time at one-half of the maximal amplitude following the peak. Area under curve (AUC) (F*s/F_0_), which is the discrete right-sided Riemann sum of the signal area during the period that is above one-half of the maximum signal amplitude (Francis et al., 2012, 2014, 2016; Shen et al., 2018).

Within the recordings from basilar arterial myocytes several different types of calcium events were observed that could be discriminated based on their temporal signaling characteristics, which were also observed in our previous studies performed in pulmonary arteries (Shen et al., 2018). These include rapid events that were between 0 and 4 s and that were scored as calcium sparks, medium duration oscillations that were defined as being between 4 and 40 s, and finally long duration oscillations that were defined both by being 40 + s and by their unique plateau at their peak fluorescence (Shen et al., 2018). Visual analysis of each data record was used to clarify the divisions between groups and to remove false positives. The number of cells with Ca^2+^ oscillations were based on visual examination of myocytes in 1,002 μm^2^ regions of interest with replicates performed in three separate regions per recording for each animal (Hadley et al., 2012; Shen et al., 2018).

### Spatiotemporal Ca^2+^ Signaling Analysis

Spatiotemporal characteristics of the whole-cell Ca^2+^ oscillatory events were analyzed by performing a cross correlation analysis in time and space for the regions of interest detected by LCPro (Shen et al., 2018). The program is utilized to gain a deeper understanding of local and distant signaling networks within smooth muscle cells as well as to better determine the methods of Ca^2+^ trafficking between cells. This program combines a custom set of physiology analysis tools written within python that enable us to compare and observe correlative Ca^2+^ oscillations within a data set. A correlation coefficient of *r* = 0.8 was used for all data sets based on parameter space searches for correlating oscillations utilizing *r* values ranging from *r* = 0.1–1.0 (Shen et al., 2018). “Friends” were determined to be calcium transients that had a correlation coefficient greater than *r* = 0.8. “Neighbors” were defined as calcium transients occurring in nearby myocytes. A radius of 100 pixels (∼23 μm) was chosen as the critical spatial cutoff because this distance allowed for the inclusion of most all neighboring cells.

### Line Scan Ca^2+^ Recordings and Analysis

Fluo-4 AM loaded basilar arterial myocytes were recorded with a Zeiss LSM 710 NLO laser scanning confocal imaging workstation on an inverted platform (Zeiss Axio Observer Z1). After being loaded with Fluo-4, the arteries were prepared and imaged as detailed above. Line scan images were captured at 529 lines per second and recordings were made for a duration of 18.9 s. The lateral pixel size ranged from 0.0148 to 0.0911 μm per pixel and the pinhole was adjusted so that the cells were imaged to a depth of 2.5 μm, which is about 50% of the width of a myocyte based on prior morphological studies in live cells (Hadley et al., 2012; Shen et al., 2018).

Line scan recordings were analyzed for the percentage of cells with Ca^2+^ sparks, their frequency, amplitude, and other spatiotemporal characteristics via *SparkLab* 4.3.1 (Harraz et al., 2015; Shen et al., 2018). Before analysis, background fluorescence was subtracted from each recording assuming a homogenous level of background noise, and the fluorescence recordings normalized (Harraz et al., 2015; Shen et al., 2018). The threshold for spark detection was set to 2 standard deviations above this background level of fluorescence. 3–4 animals were used per group for both subcellular Ca^2+^ spark and whole-cell Ca^2+^ oscillation analysis. Refer to the figure legends for specific *n* values associated with each set of data.

### Experimental Protocol

Fluo-4 loaded basilar arteries placed *en-face* in the imaging chamber were handled akin to our previous imaging studies of cerebral, pulmonary and uterine arteries (Hashad et al., 2017; Shen et al., 2018; Hu et al., 2020). Once a region of interest was identified in the control solution a video recording was made followed by line-scan images of individual myocytes in the arterial wall. These recordings required roughly 1 h. Upon completion of the video and line-scan recordings, the imaging chamber was removed from the microscope workstation and placed on the platform of a stereomicroscope. The control bathing solution was then gently removed with a pipette, and replaced with a 30 mM K bathing solution. To ensure that the bathing solution was fully exchanged the procedure was performed a total of three times. The tissues were then allowed to recover for a minimum of 5 min before resuming imaging to ensure that the cytosolic calcium reached a dynamic equilibrium.

### Drugs and Chemical Reagents

Unless otherwise indicated, all reagents were purchased from Sigma-Aldrich (St. Louis, MO, United States).

### Statistical Methods

Data analysis and the production of graphs was performed using GraphPad Prism 9.1.2 (La Jolla, CA, United States). The graphs present the data as means ± SD. Continuous variable data was tested for normality prior to analysis. These datasets were not normally distributed and as such they were evaluated using non-parametric statistical tests. A Kruskal–Wallis ANOVA with a Dunn’s multiple comparisons test was performed on non-parametric datasets when making examinations between the experimental groups based on animal age, altitude, and treatment condition. The density of cell firing in video recordings was evaluated by drawing 3 boxes of a known size in each recording and counting the number of cells with events inside the box, with each cell only being counted once. For calcium spark analysis the numbers of cells were evaluated for those cells with or without events. The number of cells in videos or lines-scan recordings that had events was summarized. Statistical comparisons of this discrete data was made by performing contingency analysis between groups using Chi-Square analysis to evaluate potential changes in the incidence of Ca^2+^ oscillatory and spark events in whole-cell and line-scan recordings. A *P*-value of *P* < 0.05 was considered significant and were further broken down to *P* < 0.01 and *P* < 0.001 where appropriate.

The specific test performed for each data set are depicted in the figure legends. Sample sizes were determined by several different measurements including the number of animals studied, the number of regions of interest showing Ca^2+^ oscillations, the number of line scans examined for Ca^2+^ sparks, and number of Ca^2+^ events. The percentage of cells firing with Ca^2+^ sparks was determined by the number of line scans containing Ca^2+^ events through visual observation relative to the total number of line scans observed.

## Results

### Whole-Cell Ca^2+^ Oscillations in Basilar Arterial Myocytes

**FIGURE 1:**
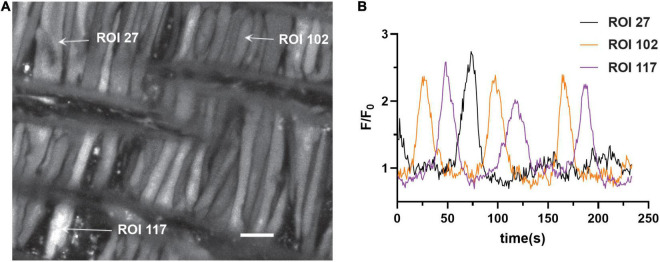
Representative Ca^2+^ responses in basilar arterial myocytes from an adult sheep recorded *en face* under 30K conditions. **(A)** maximum intensity projection for Fluo-4 fluorescence of recorded cells using laser scanning confocal microscopy. Arrows point to regions of interest in individual myocytes shown in **(B)**; fluorescence intensity tracing showing spontaneous Ca^2+^ oscillations in two ROIs. Scale bar (white) = 10 μm.

The first series of studies were designed to test the hypotheses that membrane depolarization would increase Ca^2+^ oscillatory activity and that long-term hypoxia (LTH) would impair oscillatory events in basilar arterial myocytes. Figure 1A shows a maximum intensity projection of Fluo-4 fluorescence in myocytes of the basilar arterial wall from a time series recording. A video corresponding to this image is provided in Supplementary Material Video 1. The image illustrates that the myocytes are spindle shaped and closely associated with one another and consistent with myocytes from other vascular beds and species that we have examined, including pulmonary and uterine arteries of sheep as well as cerebral arteries of rat and mesenteric arteries of mouse (Hadley et al., 2012; Harraz et al., 2014, 2015; Shen et al., 2018; Hu et al., 2020). Figure 1B shows the Fluo-4 fluorescence over time recorded from two regions of interest (ROI’s) in individual myocytes that were automatically detected with *LCPro* (Francis et al., 2012; Shen et al., 2018), a custom analysis program and subsequently analyzed for spatial and temporal aspects to the Ca^2+^ signals. These “medium duration” events were the most common in the whole-cell Ca^2+^ recordings, with the oscillations spreading relatively uniformly through the myocytes and lasting between 4 and 40 s.

**FIGURE 2:**
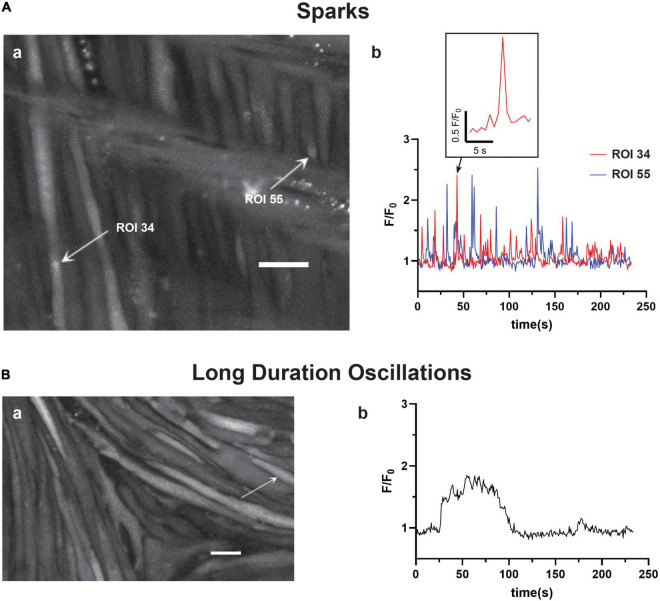
Basilar arterial myocytes show additional distinct forms of Ca^2+^ signals. The figure shows (a) maximum intensity projections with arrows pointing to regions of interest that correspond to the fluorescence intensity tracings shown in (b). This includes **(A)** short duration Ca^2+^ “spark” events (0–4 s) and **(B)** long duration Ca^2+^ oscillations (40+s). The labeled time-series tracings of F/F_0_ for these two types of Ca^2+^ responses were recorded using Fluo-4 and then detected and evaluated *post hoc* with LCPro. Scale bars (white) in (Aa and Ba) = 10 μm.

**FIGURE 3:**
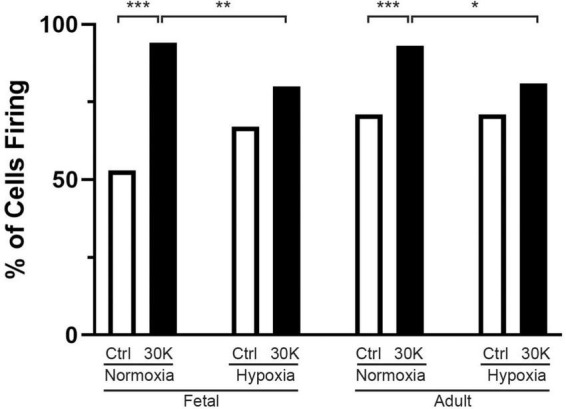
Long-term hypoxia reduces the percentage of basilar arterial myocytes with depolarization mediated Ca^2+^ oscillations in fetal and adult sheep. Each bar represents the percentage of cells with Ca^2+^ oscillations under control (clear) or with treatment of 30K (black) based on an examination of myocytes in 1,002 μm^2^ regions of interest. Replicates were performed in 3 separate regions per recording for fetal normoxic (3 animals along with 79 control and 71 30K myocytes), fetal hypoxic (4 animals with 91 control and 74 30K myocytes), adult normoxic (3 animals with 69 control and 68 30K myocytes), and adult hypoxic (4 animals with 61 control and 68 30K myocytes) animals. *P < 0.05, **P < 0.01, ***P < 0.001 indicate significance based on a chi-square test.

**FIGURE 4:**
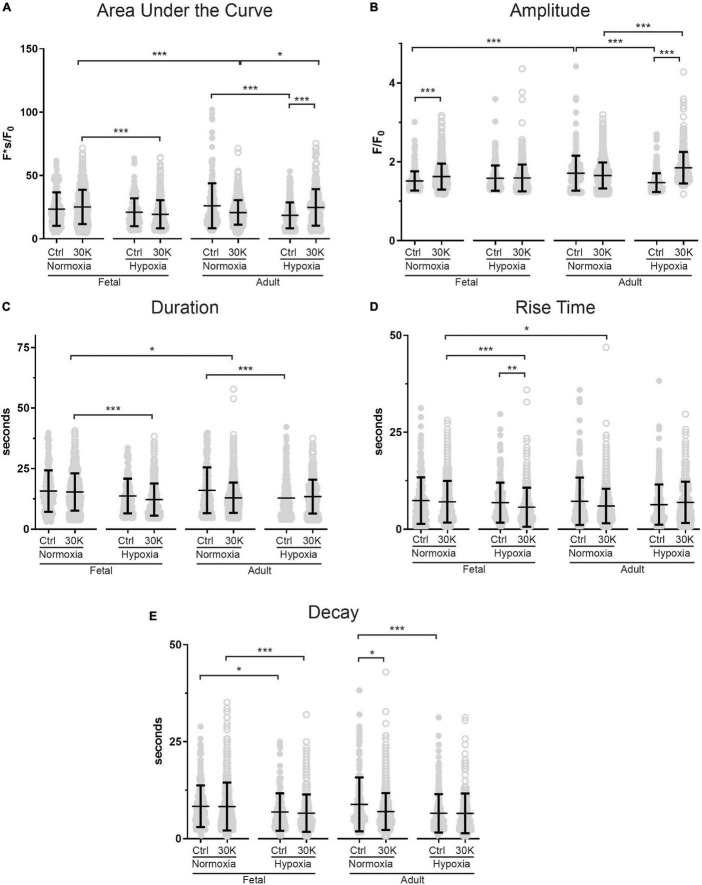
Long-term hypoxia has minimal effect on the magnitude or temporal aspects of medium duration Ca^2+^ oscillations in fetal and adult sheep. Effects of membrane depolarization with 30 mM K, long-term hypoxia, and animal age on **(A)** area under the curve, **(B)** amplitude of the fractional fluorescence, **(C)** duration of the event, **(D)** rise time, and **(E)** decay time in cytosolic fractional fluorescence. Bars represent mean ± SD for each parameter; closed circles specify control conditions, and open circles treatment with 30K. *P < 0.05, **P < 0.01, ***P < 0.001 indicate significance based on a Kruskal–Wallis ANOVA with a Dunn’s multiple comparisons test based on ranks. Control recordings were made in 207/3 FN, 201/4 FH, 225/3 AN, and 325/4 AH ROIs and animals, respectively. 30K recordings were made in 642/3 FN, 394/4 FH, 1204/3 AN, and 312/4 AH ROIs and animals, respectively.

**FIGURE 5:**
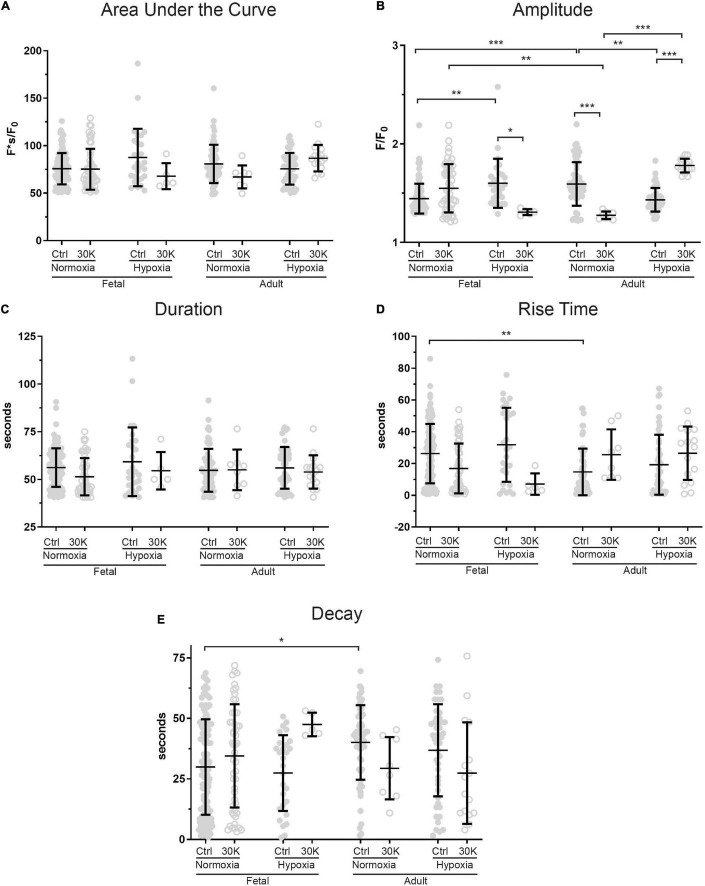
Long-term hypoxia has minimal effect on the magnitude or temporal aspects of long duration Ca^2+^ oscillations in fetal and adult sheep. Effects of artificial membrane depolarization with 30 mM K, long-term hypoxia, and animal age on **(A)** area under the curve, **(B)** amplitude of the fractional fluorescence, **(C)** duration of the fluorescent event, **(D)** rise time for fractional fluorescence in the cytosol and **(E)** decay time for cytosolic fractional fluorescence. Bars represent mean ± SD for each parameter; closed circles specify control conditions, and open circles treatment with 30K. *P < 0.05, **P < 0.01, ***P < 0.001 indicate significance based on a Kruskal–Wallis ANOVA with a Dunn’s multiple comparisons test based on ranks. Control recordings were made in 116/3 FN, 28/4 FH, 61/3 AN, and 48/4 AH ROIs and animals, respectively. 30K recordings were made in 52/3 FN, 5/4 FH, 8/3 AN, and 16/4 AH ROIs and animals, respectively.

**FIGURE 6:**
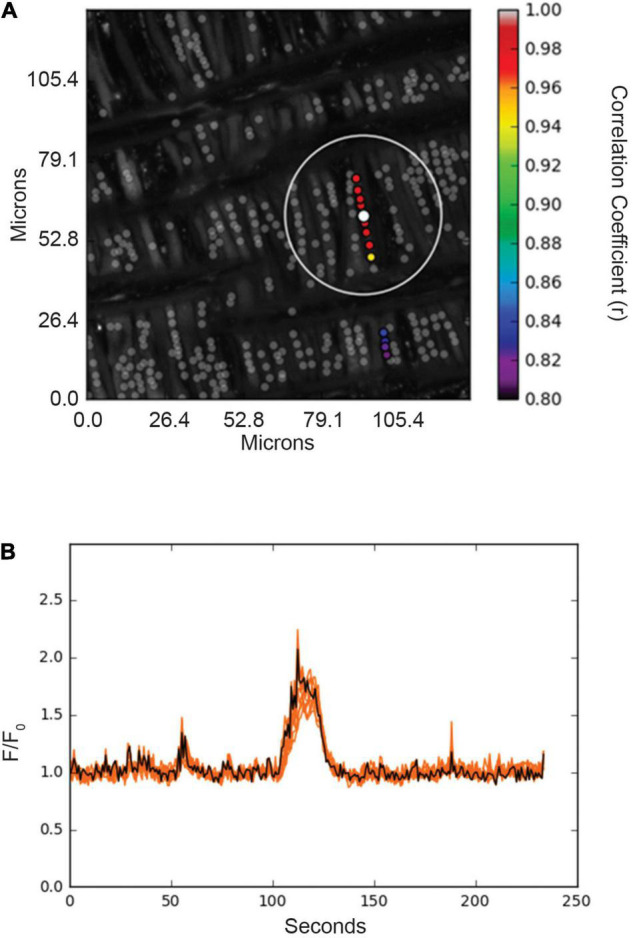
Spatial and temporal correlations of Ca^2+^ responses in basilar arterial myocytes from an adult sheep recorded *en face* under basal conditions. **(A)** Maximum intensity projection for Fluo-4 fluorescence of recorded cells using laser scanning confocal microscopy. Highly correlated events are colored and plotted around the center (reference) region of interest (ROI, white dot). Gray dots show ROIs of spontaneous Ca^2+^ oscillations that showed less than an 80% temporal correlation with the white ROI. The large white open circle shows a ∼23 μm boundary that was used for deriving spatial correlation measurements shown for the spatial and temporal analysis in Figure 7. **(B)** Fluorescence intensity tracing showing spontaneous Ca^2+^ oscillations, with the black line being the average tracing and the orange lines being the individual ROIs. ROIs were plotted using correlation coefficients with greater than 80% temporal correlation plotted surrounding the center ROI (white dot in **A**). Key shows the degree of correlation of each ROI relative to the reference ROI.

**FIGURE 7:**
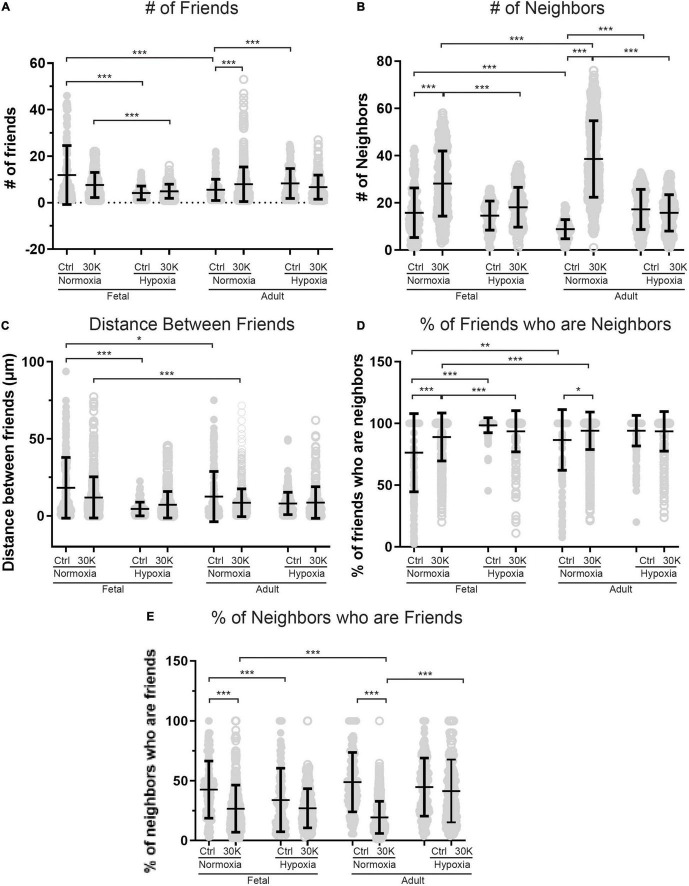
Long-term hypoxia mitigates the influence of 30K mediated membrane depolarization on friends and neighbors. **(A–E)** Arteries were analyzed for number of correlated ROIs (friends), distance between correlated ROIs, percentage of nearby ROIs (neighbors) that were correlated, and percentage of correlated ROIs that were in nearby cells. Bars represent mean ± SD for each condition; closed circles specify control conditions, and open circles treatment with 30K. *P < 0.05, **P < 0.01, ***P < 0.001 indicate significance based on based on a Kruskal–Wallis ANOVA with a Dunn’s multiple comparisons test based on ranks. Control responses were obtained from 178/3 AN, 235/4 AH, 175/3 FN, and 163/4 FH ROIs and animals, respectively. 30K responses were obtained from 844/3 AN, 244/4 AH, 537/3 FN, and 303/4 FH ROIs and animals, respectively.

**FIGURE 8:**
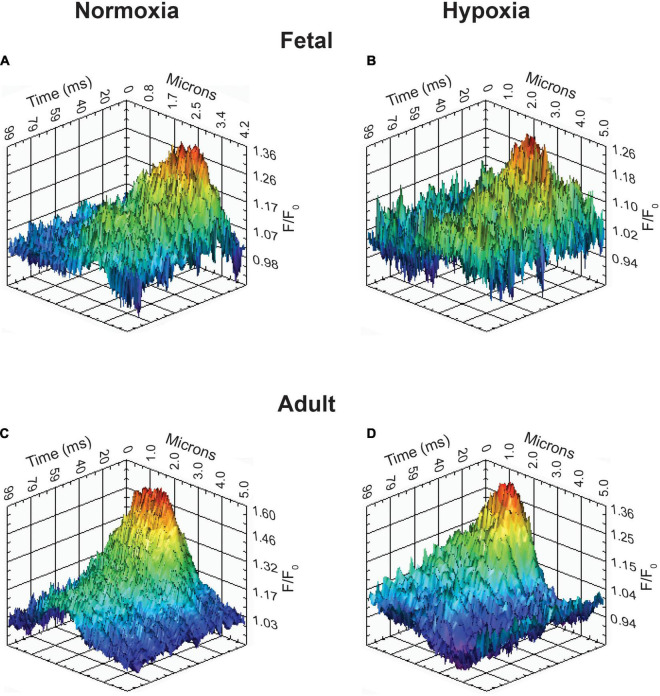
Representative Ca^2+^ spark tracings for basilar arterial myocytes from fetal and adult sheep. **(A–D)** Fluo-4 fluorescence tracings recorded from basilar arterial myocyte line scans of fetal and adult sheep under normoxic and hypoxic conditions analyzed with *SparkLab 4.3.1*.

**FIGURE 9:**
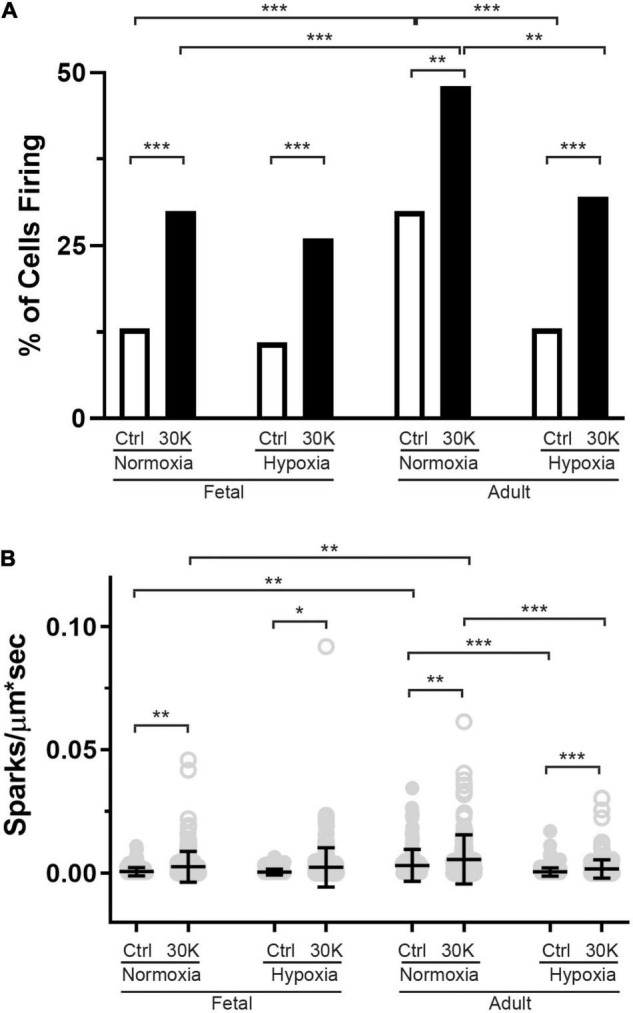
The incidence of Ca^2+^ sparks activated by membrane depolarization is reduced in fetuses and by hypoxia in adults. **(A)** percentage of cells with or without Ca^2+^ sparks and **(B)** Ca^2+^ spark firing frequency. Bars represent mean ± SD for each condition. Clear bars and closed circles specify control conditions, while black bars and open circles treatment with 30 K. Data in A were analyzed by a chi-square test while data in **(B)** were analyzed by Kruskal–Wallis one-way ANOVA with Dunn’s multiple comparison test based on ranks for each group *P < 0.05, **P < 0.01, ***P < 0.001. Recordings were made in FN control (161/3) and 30K (172/3), FH control (171/4) and 30K (185/4), AN control (150/3) and 30K (155/3), and AH control (207/4) and 30K (217/4) lines (cells)/animals, respectively.

**FIGURE 10:**
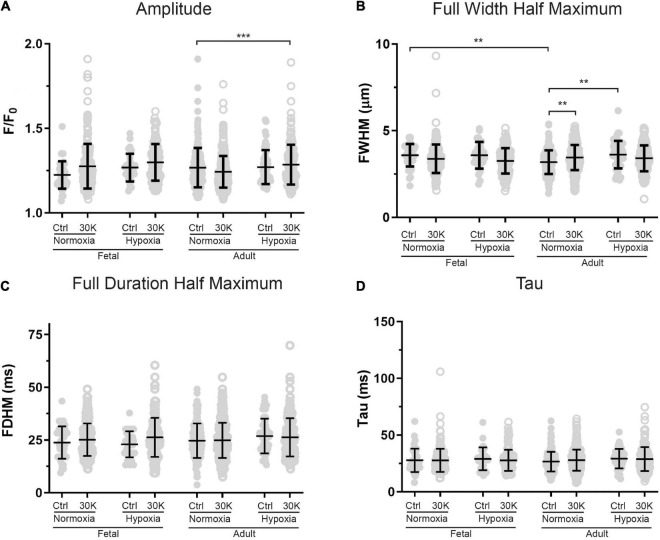
Magnitude and kinetics of Ca^2+^ sparks were minimally influenced by animal age, LTH, or membrane depolarization. **(A–D)** amplitude, full width at half-maximum, full duration at half-maximum and tau exposed to control (closed circles) or 30 K (open circles) for Ca^2+^ spark events of arterial myocytes from adult and fetal sheep under normoxic and hypoxic conditions. Bars represent means ± SD for each parameter. Data were analyzed by a Kruskal–Wallis one-way ANOVA with Dunn’s multiple comparison test based on ranks for each group **P < 0.01, ***P < 0.001. Recordings were made in FN control (41/161/3) and 30K (199/172/3), FH control (30/171/4) and 30K (136/185/4), AN control (173/150/3) and 30K (365/155/3), and AH control (52/207/4) and 30K (177/217/4) Ca^2+^ sparks/lines/animals, respectively.

The recordings of Fluo-4 fluorescence (Figure 2) show that there were a variety of different Ca^2+^ oscillatory events in addition to the medium duration events described in Figure 1, which were akin to those we identified in pulmonary arterial myocytes (Shen et al., 2018). Results from these imaging studies are presented in Figures 2–7. Figure 2 shows that some Ca^2+^ events were very rapid, and had features that were reminiscent of Ca^2+^ sparks in that they were localized in subcellular regions (Figure 2Aa) and were fast, being roughly 1–2 s in duration (Figure 2Ab), being at the edge of detectability with the full-frame recording techniques used for whole-cell Ca^2+^ recordings (Shen et al., 2018). The corresponding Supplementary Video 2 recording for Figure 2Aa illustrates that there are multiple types of Ca^2+^ events in the arterial myocytes. The inability to record rapid events with enough fidelity to perform analysis using full-frame recording techniques made at 1.28 Hz is exemplified in the exploded view of a single Ca^2+^ spark event. These rapid Ca^2+^ signaling events were scored as Ca^2+^ sparks, which are classically due to coordinated activation of ryanodine receptors on the sarcoplasmic endoplasmic reticulum (SER) that release Ca^2+^ into the cytosol (Jaggar et al., 1998a,b; Hadley et al., 2012; Hashad et al., 2017; Shen et al., 2018). These data also illustrate the need to use high-speed line-scan recordings to examine the Ca^2+^ spark events more thoroughly (Shen et al., 2018), results of which are presented in Figures 8–10.

Infrequently, we observed a third category of “long-duration” Ca^2+^ oscillations with large spatial spread through the myocytes (Figure 2Ba) and that lasted over 40 s. Figure 2Bb shows that these events had a unique quality in that they had rapid increases in cytosolic Ca^2+^ that plateaued and were sustained for an extended period followed by a prompt relaxation of the Ca^2+^ back to basal levels. The Supplementary Material Video 3 provides the recording associated with Figure 2B.

For analysis purposes, the oscillations were broken into three discrete categories: short-duration Ca^2+^ sparks, which lasted less than 4 s, medium-duration Ca^2+^ oscillations of 4–40 s, and long-duration Ca^2+^ oscillations that lasted more than 40 s (Shen et al., 2018). Acutely, the subcellular Ca^2+^ spark events are likely to regulate the activity of voltage and calcium activated potassium channels that give rise to spontaneous transient outward currents and subsequent vasodilatory responses (Jaggar et al., 1998b; Hu et al., 2011, 2012; Hashad et al., 2017). The whole-cell Ca^2+^ oscillation events in comparison are likely responsible for causing arterial contraction as well as regulating a variety of other cellular processes including metabolism or transcription (Wilson et al., 2005; Goyal et al., 2011; Papamatheakis et al., 2011; Ureña et al., 2013; Tykocki et al., 2017).

The percentage of cells with medium-duration Ca^2+^ oscillation events were impacted by animal age and LTH, with activity being enhanced by treatment with 30 mM potassium (30K), data that is summarized in Figure 3. The potassium in the extracellular bathing solution was raised to 30 mM in order to depolarize the plasma membrane and increase activation of voltage gated calcium channels. Increased calcium influx across the plasma membrane leads to more calcium in the junctional space between the plasma membrane and SER. The elevated junctional calcium subsequently activates clusters of ryanodine receptors on the SER, which gives rise to subcellular events (Ca^2+^ sparks, see Figures 8–10) and when combined with other sources of calcium there are pronounced calcium oscillations that spread through the myocyte (Jaggar et al., 1998b; Janiak et al., 2001; Tykocki et al., 2017).

The percentage of cells with calcium events was similar in the fetal and adult groups and unaffected by LTH in the control bathing solution with low (5 mM) extracellular potassium. Membrane depolarization with 30K increased calcium oscillations in normoxic fetal and adult basilar arterial myocytes, an effect that was more pronounced in adult as compared to fetal sheep. Following long term hypoxia, however, membrane depolarization failed to increase cellular calcium oscillations in both fetal and adult basilar arterial preparations.

The kinetics and magnitude of the medium-duration Ca^2+^ oscillations were then quantified, with summarized data shown in Figure 4. Overall, animal age, LTH, and membrane depolarization had only mild effects on the Ca^2+^ oscillations. Figure 4A shows that under basal conditions the area under the curve (AUC) of Ca^2+^ oscillations were unaffected by maturation. However, in adult normoxic myocytes the AUC was slightly increased relative to that in the adult hypoxic period. Membrane depolarization caused a modest increase in the AUC in adult hypoxic myocytes. The AUC was unaffected by membrane depolarization in fetal normoxic and hypoxic groups; however, the AUC was lower in myocytes from fetal hypoxic and adult normoxic relative to fetal normoxic animals following membrane depolarization. The amplitudes of the Ca^2+^ responses are shown in Figure 4B. Event amplitudes recorded from myocytes of the adult hypoxic and fetal normoxic groups were reduced relative to those from normoxic adults under basal conditions. Membrane depolarization increased Ca^2+^ event amplitude in the fetal normoxic and adult hypoxic groups. Event amplitude was also elevated in adult hypoxic relative to adult normoxic myocytes following membrane depolarization. The duration of the Ca^2+^ events is shown in Figure 4C and again there were only modest differences between the groups. There was no impact of animal age on event duration under basal conditions, however, LTH caused a shortening of the oscillatory duration in adult myocytes. Membrane depolarization had no direct influence on the oscillatory duration in any of the four groups, though fetal normoxic myocytes had longer durations as compared to events from fetal hypoxic and adult normoxic myocytes. Figure 4D shows that the Ca^2+^ rise time during the oscillations was unaffected by LTH or animal age under basal conditions. Membrane depolarization shortened the rise time modestly in only the fetal LTH group. Following membrane depolarization, the rise time was longer in fetal normoxic myocytes as compared to fetal hypoxic and adult normoxic myocytes. Figure 4E shows the decay time for the Ca^2+^ signal. Under basal conditions, LTH modestly shortened the time for the decay of the oscillatory signal in both fetal and adult myocytes. Membrane depolarization shortened the decay in myocytes from adult normoxic animals, which caused the decay duration to become equivalent with that from LTH adult animals. Following membrane depolarization, the time for Ca^2+^ decay was also shorter in LTH fetal myocytes relative to the normoxic counterparts.

### Long-Duration Ca^2+^ Oscillations

Long-duration Ca*^2+^* oscillations were significantly less common than medium duration oscillations or Ca*^2+^* sparks as illustrated by the reduced number of events. The effects of animal age, LTH, and membrane depolarization on these events are presented in Figure 5. The most notable influences were observed in the event amplitudes. Adult normoxic animals under basal conditions had increased event amplitudes (Figure 5B), decreased rise times (Figure 5D), and increased decay times relative to normoxic fetuses (Figure 5E). Figure 5B also shows that long-term hypoxia increased event amplitude in fetal animals but decreased the amplitude in adults under control conditions. Membrane depolarization decreased event amplitude in fetal hypoxic and adult normoxic animals but increased the amplitude in adult hypoxic animals.

### Spatiotemporal Ca^2+^ Signaling

The interactions of the Ca^2+^ oscillations among smooth muscle cells were then analyzed for temporal correlations between the Ca^2+^ transients, or “friendship,” between oscillations at the various ROIs. The larger the correlation coefficient, the greater the level of event “friendship.” Temporally related correlations between the Ca^2+^ events were then identified by those events that had a correlation coefficient of *r* ≥ 0.8 (Shen et al., 2018). Results from one representative sample are provided in Figure 6. Figure 6A shows an image projection with overlaid regions of interest that have varied levels of correlations, while Figure 6B shows the average fluorescence (black line) along with the fluorescence intensity values for individual regions of interest that were correlated. Spatial correlations between events are shown in Figure 7B, a relationship characterized as “neighbors” as delineated by those ROIs that were within a 100 pixels (∼23 μm) radius of any individual ROI, as delineated by the larger white circle on the image in Figure 6B (Shen et al., 2018). Animal age, LTH and membrane depolarization had diverse effects on the numbers of friends, with summary data provided in Figure 7. Figure 7 shows that relative to fetuses there was a decrease in the number of friends in adult arterial myocytes under control conditions. LTH decreased the number of friends in fetal arterial myocytes but increased them in adults under control conditions. Membrane depolarization increased the number of friends in adult normoxic myocytes. As illustrated in Figure 7B, the number of neighbors was also influenced by animal age, LTH, and membrane depolarization. Under basal conditions, there was a decrease in the number of neighbors in adults relative to fetuses. Under basal conditions, LTH increased the number of neighbors in adults but this was unaffected in fetuses. Membrane depolarization increased the number of neighbors in normoxic fetuses and adults, but LTH blunted the responses in myocytes of both age groups. Figure 7C delineates the distance of temporally correlated events (friends). The distance between friends was shorter in normoxic adults relative to fetuses under control and depolarized conditions. Long-term hypoxia resulted in a shortening of the distance only in fetuses under control conditions. Figure 7D provides the percentage of friends who are neighbors, illustrating the spatial and temporal relationships between Ca^2+^ oscillations. Overall, a majority of temporally correlated oscillatory events (friends) are closely associated (neighbors), however, there were some subtle effects of animal age, LTH, and membrane depolarization. There was a slight increase in the percentage of adult normoxic myocyte ROIs who were neighbors relative to those in fetal normoxic myocytes. Although there were fewer Ca^2+^ oscillatory events in fetal hypoxic myocytes than their normoxic counterparts, a greater proportion were neighbors under control conditions. Membrane depolarization preferentially increased the percentage of friends who were neighbors in normoxic myocytes independent of animal age. Figure 7E presents the percentage of neighbors who were friends, which provides an index of the temporal relationship between Ca^2+^ oscillatory events that are spatially related. Unlike the tight spatial relationship between temporally related events, events that occur in nearby regions are not necessarily temporally correlated. Even still, there were influences of LTH and membrane depolarization. Long-term hypoxia preferentially reduced the percentage of neighbors who were friends in fetal myocytes under control conditions. Membrane depolarization preferentially reduced the percentage of neighbors who were friends in myocytes of fetal and adult normoxic animals.

### Activation of Ca^2+^ Sparks

The last series of studies examined the impact of membrane depolarization and hypoxia on the activation of Ca^2+^ sparks in myocytes from fetuses and adults. Figure 8 shows representative Ca^2+^ spark tracings from line scan recordings for control groups analyzed with *SparkLab* (Figures 8A–D; Shen et al., 2018). Our lab and others have shown that membrane depolarization with extracellular K^+^ can enhance Ca^2+^ spark activity and vascular contractility, which is why we chose to depolarize the membrane with 30 mM K^+^ (Jaggar et al., 1998a; Hadley et al., 2012; Papamatheakis et al., 2012; Harraz et al., 2014; Shen et al., 2018). Membrane depolarization with 30K increased the prevalence of myocytes with Ca^2+^ spark activity in all groups and the frequency of activation as shown in Figures 9A,B. Under control conditions the prevalence and frequency of Ca^2+^ sparks in myocytes from normoxic adults were greater than in the fetal period. LTH did not impact the prevalence or frequency of Ca^2+^ sparks in fetuses, but reduced spark activity in adult myocytes to fetal levels. Membrane depolarization increased Ca^2+^ spark activation in adult animals while LTH preferentially blunted spark activation in adults. Overall, these findings illustrate that LTH impairs spark activity in basilar arteries but support the general influence of membrane depolarization on Ca^2+^ spark activity that we have shown in our previous studies on pulmonary and uterine arteries from sheep as well as in middle cerebral as well as mesenteric arteries from rats and mice (Hadley et al., 2012; Harraz et al., 2014, 2015; Shen et al., 2018; Hu et al., 2019).

The magnitude and kinetics of Ca^2+^ sparks were quantified in basilar arterial myocytes with the summary results shown in Figures 10A–D. Overall, there was little impact of animal age, LTH, or membrane depolarization on the quality of the Ca^2+^ sparks. When comparing events in myocytes from fetuses and adults, the full width of half maximum was narrowed slightly in normoxic control conditions. Long-term hypoxia increased the full width of half maximum of Ca^2+^ sparks in adult myocytes under control conditions. Membrane depolarization modestly increased the full width of half maximum of Ca^2+^ sparks in adult normoxic myocytes and there was no impact on the exponential decay time constant (Tau) of the Ca^2+^ spark events.

## Discussion

The current study was designed to examine the impact of animal age and high altitude induced LTH on cytosolic Ca^2+^ oscillations and sparks in basilar arterial myocytes. These studies were performed because of the integral role basilar arteries have in regulating vascular reactivity and providing blood flow to the brain stem, which is critical to autonomic cardiorespiratory functions that are impacted by LTH. Basilar arterial myocytes had robust Ca^2+^ oscillations and the number of event sites were increased by membrane depolarization. The data also illustrate that animal age and LTH modify the coupling of membrane depolarization to the activation of whole-cell Ca^2+^ oscillations as well as the generation of localized Ca^2+^ sparks.

### Whole-Cell Ca^2+^ Oscillations

Restriction of whole-cell intracellular Ca^2+^ oscillations by LTH is not unique to the cerebral vasculature. Previously, we reported that Ca^2+^ oscillatory activity was reduced by LTH in pulmonary arterial myocytes (Hadley et al., 2012; Shen et al., 2018). The parallel changes in Ca^2+^ signals in basilar and pulmonary arterial myocytes due to LTH suggest there may be commonalities in the mechanisms underlying the changes in oscillatory activity following LTH. Myocyte cytosolic Ca^2+^ oscillations are dependent on a functional SER (Mufti et al., 2010) and InsP_3_ receptor activation is important to the generation of oscillatory signals (Adebiyi et al., 2010, 2011; Mufti et al., 2015; Tykocki et al., 2017). These relationships lead to the potential that losses in Ca^2+^ oscillatory activity and other temporal signaling aspects by LTH may be coupled to impairments in InsP_3_ signaling. Indeed, previous work from our group shows that LTH reduces InsP_3_ receptor expression in both fetal and adult middle cerebral arteries (Longo et al., 1996; Ueno et al., 1997). Depression in InsP_3_ signaling may therefore also underlie the suppression in membrane depolarization induced Ca^2+^ oscillations we report here. With regards to the current findings that LTH restricts cellular Ca^2+^ responses, our previous data illustrate that the SER Ca^2+^ stores of sheep pulmonary arteries are largely intact regardless of age or hypoxic stress (Hadley et al., 2012), although there is significant SER stress as evidenced by structural changes in the SER of pulmonary arterial myocytes as well as increases in markers of SER stress (Leslie et al., 2021). Such evidence suggests that the major aberrations in Ca^2+^ responses are more likely due to dysregulation of pathways critical to regulating Ca^2+^ signaling that may involve SER stress as opposed to fundamental disruption of the storage capabilities of the intracellular Ca^2+^ stores.

Membrane depolarization dependent increases in Ca^2+^ oscillatory activity was expected as this is common in smooth muscle (Iino, 1990; Hadley et al., 2012; Shen et al., 2018). Increases in oscillatory frequency with membrane depolarization may be the result of changes in the speed of Ca^2+^ release and uptake at the SER as well as increase Ca^2+^ flux across the plasma membrane (Keizer et al., 1995; Wilson et al., 2002, 2005; Goyal et al., 2009). The increases in the oscillatory amplitude of cells from fetal animals suggests that membrane depolarization may increase oscillatory signals either through increased activation of InsP_3_ or ryanodine receptors on the SER or through enhanced recruitment and coupling to L-Type Ca^2+^ channels (Jaggar et al., 1998b; Blood et al., 2002; del Corsso et al., 2006; Ureña et al., 2013) or other Ca^2+^ permeable plasma membrane ion channels (Jaggar et al., 1998b; Janiak et al., 2001; Earley et al., 2005; Adebiyi et al., 2010, 2011; Dahan et al., 2012; Ureña et al., 2013; Harraz et al., 2014). In this regard, the picture that emerges is that the impact of LTH on basilar arterial Ca^2+^ signaling is complex and mediated through modification of multiple pathways important to the regulation of intracellular Ca^2+^.

The finding that whole-cell Ca^2+^ oscillations were reduced in basilar arterial myocytes from normoxic fetuses compared to adults builds from our previous evidence of age-related changes in Ca^2+^ signaling of cerebral arteries (Ducsay et al., 2018). One possibility is that these differences are coupled to developmental related effects on the role of SER and Ca^2+^ influx pathways to contractility. Indeed, contractions of cerebral arteries from fetal sheep have reduced reliance on release of intracellular Ca^2+^ stores and increased dependence on Ca^2+^ influx as compared to adults (Long et al., 2000). These changes in arterial contractions were shown to be coupled to greater L-type Ca^2+^ channel protein expression in fetus and responsiveness to L-type Ca^2+^ channel activation (Long et al., 2000; Blood et al., 2002). Even still, Ca^2+^ oscillations are due to sequential filling and release of internal Ca^2+^ stores in smooth muscle, with modulation through extracellular Ca^2+^ influx pathways (Janiak et al., 2001; Wilson et al., 2005; Hume et al., 2009). Given that we did not fully assess the role of extracellular Ca^2+^ entry or release of intracellular Ca^2+^ stores to the oscillatory activity in basilar arterial myocytes, we still do not know what role age has to the process of Ca^2+^ handling.

The influence of membrane depolarization on the temporal and spatial aspects to the Ca^2+^ oscillations in normoxic animals are compelling. When the tissue was depolarized, there were significantly more events during the recordings in the arterial myocytes as demonstrated by the uptick in spatially related events (neighbors), however, there were fewer temporally related events (friends). On closer examination the neighboring events mainly occurred within individual cells as opposed to adjacent cells. While we have not explored this deeply in the current study, the findings suggest that when myocytes are depolarized the Ca^2+^ oscillations do not propagate from cell to cell, but rather, the number of events increase through recruitment of unrelated cells across the arterial wall. One explanation for this apparent lack of cell-to-cell communication is that membrane depolarization recruits additional L-Type Ca^2+^ channels on individual cells in a stochastic manner, which then increases Ca^2+^ responses in separate cells. Further, the data suggest gap junction connections between myocytes are not being activated, which limits cell-to-cell propagation of Ca^2+^ signals (Hald et al., 2014; Welsh et al., 2018; Zechariah et al., 2020). Such findings are not unfounded as physiological studies and computational models suggest there is poor electromechanical coupling between arterial myocytes but propagation is spread more easily through the vascular endothelium (Hald et al., 2014; Tykocki et al., 2017; Welsh et al., 2018).

### Ca^2+^ Spark Activity

Previous evidence illustrates that the localized rapid release of calcium, coined Ca^2+^ sparks, in cerebral vascular myocytes is due to coordinated activation of ryanodine receptor clusters (Jaggar et al., 1998a,b). These Ca^2+^ spark events are important as they are coupled to vasodilation through activation of large conductance potassium channels (BK_Ca_). Depolarization of the plasma membrane is well regarded to enhance Ca^2+^ spark activity and the ensuing repolarization of the membrane and vasodilation acts as a negative feedback regulator of vasoreactivity. With regards to the effects of age and LTH, basilar arterial myocytes from adults had far greater Ca^2+^ spark activity relative to those from fetuses. Furthermore, LTH reduced spark activity in adults to fetal levels and suppressed the depolarization mediated increase in Ca^2+^ spark activity in adult myocytes. The inability of depolarization to increase Ca^2+^ spark activity in adults following LTH is interesting and reminiscent of the impact of LTH on fetal pulmonary arterial myocytes (Hadley et al., 2012; Shen et al., 2018). Much like the impact on whole-cell Ca^2+^ oscillations, this parallel effect of LTH on basilar and pulmonary arterial myocytes suggests there may be a common underlying mechanism whereby LTH suppresses depolarization induced activation of Ca^2+^ sparks. Presumably, this would be due to a loss in communication between plasma membrane channels and ryanodine receptors on the SER, possibly involving SER stress such as we have shown in fetal pulmonary arterial myocytes (Leslie et al., 2021). These losses in communication potentially include L-type Ca^2+^ channels but may also involve disruption in T-type Ca^2+^ channels or TRPV4, which are also important to Ca^2+^ spark activation in vascular myocytes (Earley et al., 2005; Harraz et al., 2014, 2015).

The impact of age and long-term hypoxia on the role of the Ca^2+^ spark events to feedback activation of BK_Ca_ is not fully resolved. Previous evidence from our group, however, illustrates that BK_Ca_ from fetal basilar arteries have increased activity in response to changes in the cytosolic Ca^2+^ (Lin et al., 2003) mediated through enhanced protein kinase G (Lin et al., 2005, 2006). Further, LTH increases membrane expression of BK_Ca_ and Ca^2+^ dependent channel activity in both fetal and adult basilar arterial myocytes (Tao et al., 2015). Together these data suggest that even though fetal basilar myocytes have reduced Ca^2+^ spark activity relative to adults there may be preservation of BK_Ca_ channel activity and vasodilatory capacity through altered channel regulation. Similarly, vasodilation may be maintained following LTH in adults even though there is suppression in Ca^2+^ spark activity through enhanced Ca^2+^ dependent activation of BK_Ca_. Unraveling the full impacts of age and LTH on the regulation of basilar arterial reactivity and the influence this has on brain blood flow will require further interrogation.

### Perspectives

The impact of long-term hypoxia on cerebral vascular function remains poorly understood, especially with regards to the differing influences on fetuses and adults. The current data begin to shed light on the complexity of the effects of LTH on cellular Ca^2+^ signals depending on animal age that are important to vascular reactivity. The adaptations in Ca^2+^ signaling to LTH reported here are potentially juxtaposed by modifications in the regulation of BK_Ca_ channels, which are critical components in the feedback regulation of vascular reactivity and brain blood flow. These effects lead us to speculate that LTH-induced dysregulation in vascular function prompts compensatory responses that act to preserve brainstem nutrient and oxygen delivery.

## Data Availability Statement

The raw data supporting the conclusions of this article will be made available by the authors, without undue reservation.

## Ethics Statement

The animal study was reviewed and approved by the Loma Linda University Health Institutional Animal Care and Use Committee. All study procedures adhered to the Animal Welfare Act, the National Institutes of Health Guide for the Care and Use of Laboratory Animals (https://grants.nih.gov/grants/olaw/Guide-for-the-Care-and-use-of-laboratory-animals.pdf), “The Guiding Principles in the Care and Use of Animals” approved by the Council of the American Physiological Society.

## Author Contributions

SW, CW, AB, and LZ contributed to conception and design of the study. MR and NO performed laboratory experimentation. CW and JLP designed customized software for data analysis. SBC, MR, NO, and CR performed data and statistical analysis. CR wrote the first draft of the manuscript. SC wrote sections of the manuscript. All authors contributed to manuscript revision, read, and approved the submitted version.

## Conflict of Interest

The authors declare that the research was conducted in the absence of any commercial or financial relationships that could be construed as a potential conflict of interest.

## Publisher’s Note

All claims expressed in this article are solely those of the authors and do not necessarily represent those of their affiliated organizations, or those of the publisher, the editors and the reviewers. Any product that may be evaluated in this article, or claim that may be made by its manufacturer, is not guaranteed or endorsed by the publisher.
